# Making the Bend: DNA Tertiary Structure and Protein-DNA Interactions

**DOI:** 10.3390/ijms150712335

**Published:** 2014-07-14

**Authors:** Sabrina Harteis, Sabine Schneider

**Affiliations:** Department of Chemistry, Technische Universität München, Lichtenbergstrasse 4, 85747 Garching, Germany; E-Mail: sabrina.harteis@gmail.com

**Keywords:** DNA structure, DNA structure recognition, protein-DNA interactions

## Abstract

DNA structure functions as an overlapping code to the DNA sequence. Rapid progress in understanding the role of DNA structure in gene regulation, DNA damage recognition and genome stability has been made. The three dimensional structure of both proteins and DNA plays a crucial role for their specific interaction, and proteins can recognise the chemical signature of DNA sequence (“base readout”) as well as the intrinsic DNA structure (“shape recognition”). These recognition mechanisms do not exist in isolation but, depending on the individual interaction partners, are combined to various extents. Driving force for the interaction between protein and DNA remain the unique thermodynamics of each individual DNA-protein pair. In this review we focus on the structures and conformations adopted by DNA, both influenced by and influencing the specific interaction with the corresponding protein binding partner, as well as their underlying thermodynamics.

## 1. The Overall Topology of DNA

**Figure 1:**
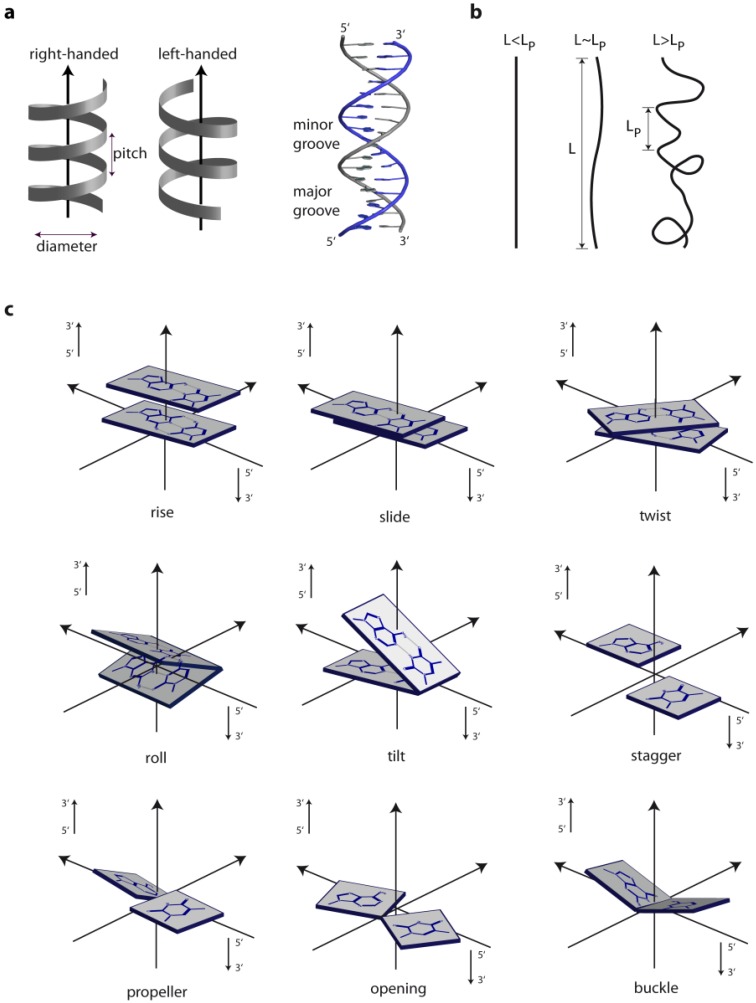
Geometric and physical parameters of DNA. (**a**) Helix parameters; (**b**) The persistence length (L_P_) provides a measure of the rigidity of a linear polymer and is determined by the change in orientation of a polymer backbone as its chain contour is traversed. A greater chain conformational freedom is reflected by a shorter persistence length; (**c**) Examples for geometric parameters defining the location and orientation of the base pair steps [9,10,11,12].

**Figure 2:**
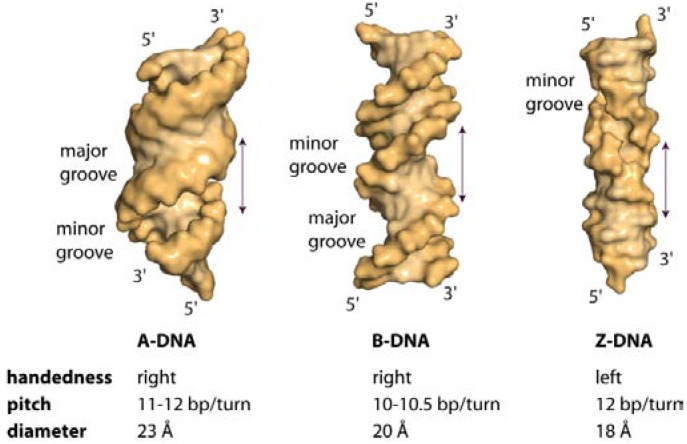
Surface representation of A-, B- and Z-DNA. Hexadecameric A- and B-DNA helices with alternating (ATGC)_4_ sequence were generated in COOT [13]. Z-DNA with the sequence (GC)_8_ was constructed with 3DNA [14]. A-DNA is slightly unwound, possessing a shallow, wide minor groove and a deep, narrow major groove. B-DNA features a narrow minor groove and a broad major groove. In the left-handed Z-DNA, named by the zig-zag pattern in the phosphodiester backbone, a deep minor groove is observed, while the major groove is reorganised and features a convex surface.

The three different general topologies taken up by the DNA double helix are termed A, B and Z. These conformations are distinguished by the handedness of the helix, their pitch (the distance between base and the base obtained after a full 360° turn), the number of nucleotides within one pitch and the distance between consecutive bases (the rise) [1]. Figure 1 schematically depicts the physical as well as the geometric parameters defining the location and orientation of the base pair steps relative to each other in the DNA helix. While RNA double strands all exist in the A-conformation, DNA adopts mainly the B-conformation (Figure 2a,b), with both forming right-handed helices. However, DNA double strands are able to take up the A-conformation in some protein-DNA complexes and under dehydrated conditions [2,3]. DNA-RNA hybrid duplexes adopt conformations between A- and B-type duplex geometry, with the RNA strand having an overall A-type structure and the DNA strand a structure intermediate of A- and B-type [4]. Depending on the nucleic acid sequence and binding partner, conformational changes to either A- or B-type duplexes are possible [5,6]. In the A-form helix, the ribose is puckered in the C3' endo, which in dsRNA is caused by the steric hindrance of the C2' endo puckering by the sugar 2'hydroxl group. In contrast, in the B-form, C2' endo ribose is found. This difference in sugar puckering results in a reduced P-P distance of 5.9 Å in the A-conformation compared to 7.0 Å in the B-conformation, as well as a shortening of the distance between the stacked bases (B-conformation: 3.30–3.37 Å; A-conformation: 2.59–3.29 Å). In addition, the A-form helix is slightly unwound with 11–12 nucleotides for every 360° turn, while in B-DNA there are 10–10.5 nucleotides. The helix axis runs almost straight through the centre of the base pair in the B-form, while in the A-form the centre of the base pairs are shifted about 4.5 Å from the axis. Thus the B-helix features two grooves, the major and the minor grooves, which differ in their width but are equally deep. (Figure 2) In contrast, the A-form helix possesses a small but deep major groove, only accessible to water and metal ions, and a shallow, but wide minor groove. (Figure 2) The structural properties of the DNA grooves strongly influence the recognition and interaction with the protein partners that will be discussed below.

The term Z-DNA stems from the observed zig-zag conformation of the phosphate backbone of a left-handed helix taken up by alternating purine-pyrimidine DNA sequences (GC repeats) under high salt conditions [7]. Here, due to the displacement of the base pairs away from the axis, only one groove can be observed that is analogous to the minor groove of B-DNA. The bases forming the major groove in B-DNA are reorganized in Z-DNA as such that they build a convex outer surface. (Figure 2c) Each guanine base is rotated around the glycosidic bond into *syn*-conformation, with the sugar puckered in C3' endo-conformation, while the cytosine in the adjacent base step is in the C2' endo, *anti*-conformation. Compared to A- and B-DNA, Z-DNA possesses a helical diameter of 18 Å, with 12 bp per helical turn, a rise of 3.7 Å and rotation by 30° per bp. In addition, the phosphate groups are closer together. Due to the electrostatic repulsion of the phosphate groups and the energy penalty associated with rotation of the Gs into *syn*-conformation, under physiological conditions the Z-conformation is the less favoured higher energy state and the DNA is pushed into the B-form. This also explains why Z-DNA becomes the stable conformation under high-salt concentrations, since the salt decreases the electrostatic repulsion of the phosphate [1,8].

## 2. DNA Sequence and DNA Structure

**Figure 3:**
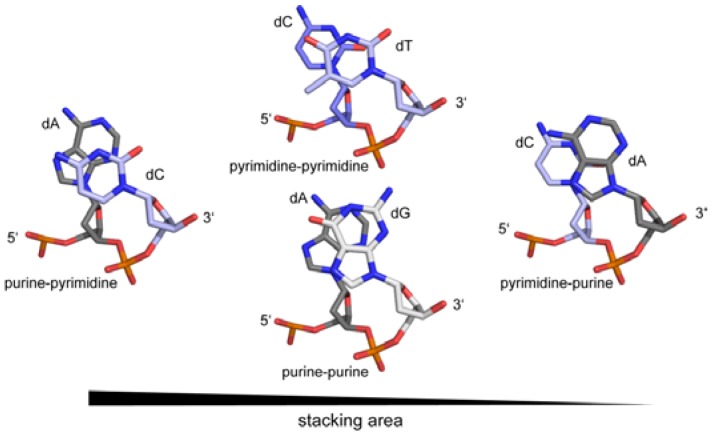
Direct correlation of the stacking area of base steps in DNA and their stiffness.

In recent years, it became apparent that the genome contains additional information to the triplet genetic code. Within and outside protein coding regions, overlapping to the genetic code, DNA shape plays a crucial role in gene regulation, genome organisation and integrity [15,16,17]. It was postulated that proteins recognise and use the intrinsic flexibility of the DNA [18,19]. Nevertheless, the sequence of the DNA is an essential prerequisite that a certain structure can be adopted. Here, the electronic configurations of the base pairs, the number of H-bonds and the presence of exocyclic groups in the major and minor grooves not only determine the deformability of the DNA, but also the deformation energy necessary to adopt a particular conformation [20,21,22]. For example, the melting temperature of any given DNA duplex is dependent on the number of H-bonds in the base pairs (GC *versus* AT). Moreover the sequence also influences the persistence length (the quantitative term for the stiffness of a polymer) of the DNA, which is directly linked to the base pair rigidity. A/T rich sequences have a lower persistence length and are therefore more bendable than G/C rich sequences [23]. Another factor impacting the DNA rigidity is the stacking interactions of the base pairs themselves. A purine-pyrimidine base step is thermodynamically more stable due to the larger stacking area than purine-purine or pyrimidine-pyrimidine base steps, with the pyrimidine-purine being the least stable. (Figure 3) Thus the persistence length of the DNA is determined by the sequence as well as its composition and context, with the stacking energy being the major factor for the base step stability. The deformability of a given DNA strand, which is defined as the range of conformational space that can be adopted, is inversely related to its persistence length. Thus sequences with high melting and stacking energy (AC/GT, GC and GA/TC) are on average less deformable compared to sequences with low melting and stacking energy (*i.e.*, AA/TT, AT and TA) [24,25,26,27]. Investigations on the flexibility of DNA strands with biological relevant length (5–100 nm~15–300 bp) by atomic-force microscopy have shown that the probability of spontaneous sharp bending is higher than predicted. Thus the bendability cannot be extrapolated from long-length-scale measurements and local effects like the DNA sequence must be taken into account [28]. Nevertheless electrostatic and nonelectrostatic effects play a role in determining the stiffness of DNA [29]. In addition, geometric analysis of available DNA structures showed that on a base-step level many conformers exist that cannot be classified as either A- or B-type, and/or represent an A-to-B transitional state [30].

**Figure 4:**
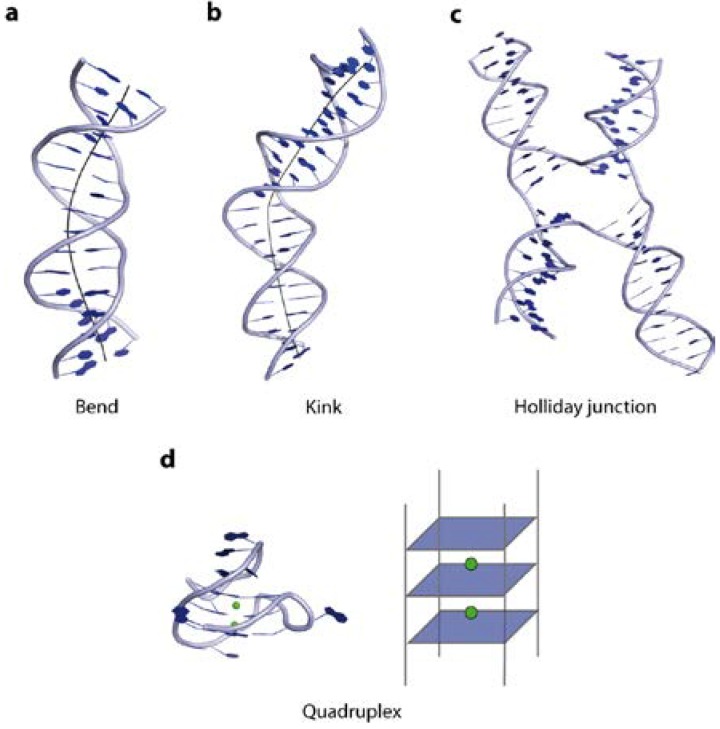
Illustration of DNA tertiary structures. (**a**) Curvature of the double helix over several bases results in a DNA bend (PDB code 1JJ4); (**b**) A DNA kink causes a change of strand orientation in an otherwise linear double strand (PDB code 2KEI); (**c**) Holliday junctions are formed by strand exchange between two DNA double helices (PDB code 2QNC); (**d**) Quadruplex DNA, a four-stranded structure, consists of guanine rich sequences, harbouring metal ions (highlighted in green) in the centre (PDB code 3QXR).

In the following paragraphs, we illustrate the structural variations taken up by the DNA, like Z-DNA conformation, bends, kinks and intriguing tertiary higher order structures, such as quadruplexes and cruciforms, and their underlying sequence dependency (Figure 4).

**Figure 5:**
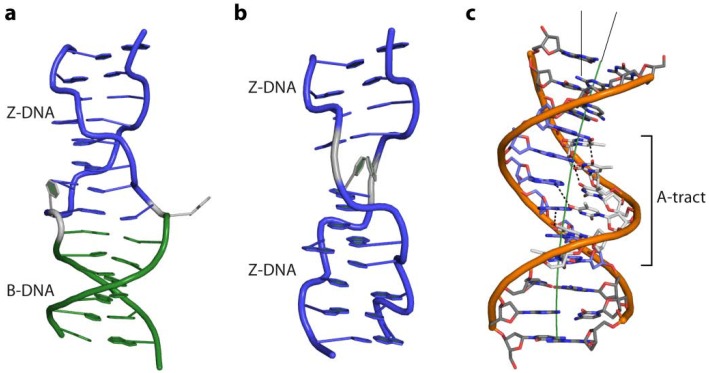
(**a**) Cartoon representation of a BZ-junction (PDB code 2ACJ) and (**b**) a Z-Z junction (PDB code 3IRR); (**c**) Intrinsic curvature of an A-tract containing DNA (PDB code 1D98) due to the propeller twist of its base pairs. Non-Watson-Crick H-bonds, between the base steps in the major groove, which rigidify the DNA strand, are shown as dashed lines.

As already mentioned above, GC repeats undergo a B- to Z-conformation transition in the presence of high salt concentrations. Next to the structural B-to-Z transition point, the so-called B-Z-junction, the helical parameter corresponds to standard B- and Z-DNA (Figure 5a). Interruption of the dinucleotide repeats by single base insertions or deletions brings neighbouring helices out of phase, and a Z-to-Z junction is formed, requiring less energy than a B-Z-junction [31] (Figure 5b). In genomes long stretches of GC repeats are rare since they represent hot spots of instability [32]. *In vivo* Z-conformations of DNA are found in regions of supercoiled B-DNA near the promoter regions where they stimulate transcription [1,33,34]. It was shown that DNA is negatively supercoiled in Z-conformation when it becomes unwrapped from histones. This prevents reformation of the nucleosome, since Z-DNA cannot form nucleosomes and the site remains free for transcription factors to bind and initiation of transcription [8,35]. Moreover, during transcription the DNA behind the moving RNA polymerase is unwound and subjected to negative torsional strain [36] further stabilising Z-DNA formation near the transcription start site. A number of proteins specifically interacting with Z-DNA are known to date: the fish kinase PKZ, the innate immune system receptor ZBP1 (also known as DLM-1 and DA), the pox-virus inhibitor of interferon (IFN) response E3L and the IFN-induced form of the RNA editing enzyme ADAR1. They are all involved in processes related to the IFN system, important for defending the organism against viruses, microbes and tumour cells [37,38,39,40,41].

Bent DNA is curved over a stretch of several bases resulting in different orientation of the regions on both sides of the curvature (Figure 4a). It was shown that short repeated stretches of poly-TA sequences or repetitive runs of 4–6 adenine base pairs (“A-tracts”) introduce an intrinsic curvature of the helix [27]. At the same time they increase the rigidity of the DNA strand, which contradicts the observation that AT-rich sequences are generally more flexible. The crystal structure of a poly-TA DNA explains these observed physical properties: the bases within a base pair are not coplanar but possess a propeller twist and are additionally buckled (Figure 5c). This results in a reduced helical repeat (10 base pairs per turn) and narrowing of the minor groove [42,43,44]. The structure is further stabilized and hence rigidified by non-Watson-Crick H-bonds formed down the major groove, enabled by the propeller twist of the base pairs. Bending of DNA containing such poly-TA stretches occurs due to their zero net roll. In standard B-DNA the net roll angle of each half turn are not zero and cancel each other out, resulting in a straight strand. Thus if an AT-stretch is present, the roll angle is not cancelled out causing a change in direction of the helix at the junction to the AT-stretch. (Figure 5c) [23] The increased stiffness prevents wrapping of such sequences around histones. In yeast, runs of about 20 bp of such poly-TA sequences are found upstream from promoter elements of constitutively active transcribed genes [45,46]. Moreover, the presence of guanine bases in the minor groove prevents bending of DNA due to the strong steric constraint imposed by the exocyclic 2-amino group [22]. In contrast, a kink in the DNA is defined as local unstacking of a single base pair step in an otherwise linear DNA strand, causing a change of orientation of the helix (Figure 4b). In this case the pyrimidine-purine (TA; CA) steps exhibiting the lowest stacking energy appear to act as a flexible “hinge” in DNA-protein interactions (Figure 3) [47,48,49].

**Figure 6:**
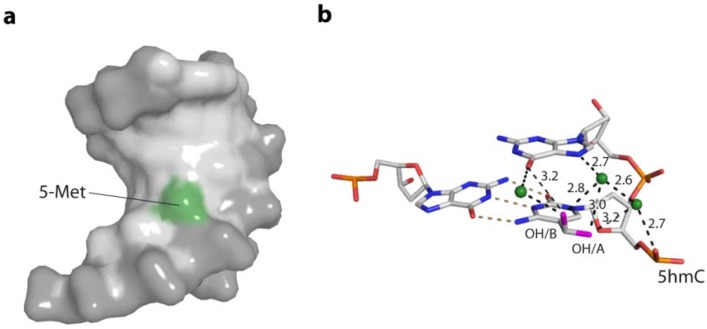
Structural bases for the increased stiffness of DNA containing the epigenetic bases 5mC and 5hmC. (**a**) The 5-methylgroup of 5mC (green) reaches in the major groove of the DNA, altering the charge distribution and steric properties in the major groove; (PDB code 4C63) (**b**) Direct and water-mediated hydrogen bonding of the two alternative hydroxyl conformations (pink) of 5hmC to the 3' base and phosphate backbone. Water molecules are depicted as green spheres. (PDB code 4C5X)

Beyond the canonical bases, epigenetic DNA base modifications, such as the methylation of cytosines (5mC) alter the anisotropic DNA bendability or flexibility at the particular site and can be linked to their regulatory effects. It was argued that the presence of 5mC rigidifies the backbone of the DNA [50] increases base pair stacking [51,52] and alters the solvation dynamics in the major groove [53]. It was also shown that 5mC changes the net curvature of A-tract containing DNA [54]. Ultimately, the presence of 5mC in CpG-islands of promoter regions affects not only the protein-binding partners [55], but also the wrapping around histones results in changes of histone positioning [21,56,57]. Recently, the influence of 5mC and hydroxymethyl C (5hmC) in DNA duplexes on the structure of the DNA and the interaction with the basic helix-loop-helix (bHLH) transcription factors Max and USF was investigated. This revealed that while no direct structural impact of the modification on the B-DNA was observed, the symmetrical presence on both DNA strands of either modified base completely abolished protein interaction, while hemi-modification was partly tolerated by the transcription factors [58]. Figure 6 shows the structural basis for the altered bendability of 5mC- and 5hmC-containing DNA, by reaching of the 5-methylgroup in the major groove of the DNA, altering the charge distribution and steric properties. Interestingly, 5mC in context of a GC repeat region facilitates the B- to Z-DNA transition [59].

The formation of higher order tertiary structures such as junctions, cruciforms and quadruplexes strongly depend on the DNA sequence. Cruciform structures, also known as Holliday junctions (Hj), occur when the four helices of two DNA duplexes are interconnected by strand exchange at a branch point, forming a 4-way, stacked X-shaped structure [60,61,62,63] (Figure 4c). All Hjs consist of a stem, a branch point and a loop, with the loop size depending on the length of the gap between the repeats. For their formation either perfect or imperfect inverted repeats of 6 bases or more are required. The gap sequence was shown to have a direct impact on Hj formation, with AT-rich sequences possessing the highest probability. In the absence of Mg^2+^ ions, Hjs have a square-planar conformation, while in their presence conformational isomer forms of stacked-X junctions are observed, all of which are in rapid exchange with each other. Nevertheless the equilibrium for a distinct conformer is biased not only by metal ions but also by the DNA sequence [64,65].

Quadruplexes, also termed tetraplexes or G4 structures, are four-stranded DNA structures, formed by guanine-rich sequences. Here, Hoogsteen-hydrogen-bonded guanines build up tetrads in a cation dependent manner [66] (Figure 4d). *In vivo*, sequences giving rise to quadruplexes have been identified in G-rich eukaryotic telomeres and promoter regions [67,68,69,70]. It was recently shown that the formation of quadruplexes in eukaryotes is modulated in the course of DNA replication [71]. In addition, sites containing G-quadruplex structures are particularly prone to DNA strand breaks and chromosomal rearrangements [72,73]. A number of proteins interacting with G-quadruplexes have been identified, all of which are involved in genome maintenance such as helicases (FANCJ, BLM), nucleosome remodelling (ATXR) or DNA damage tolerance (Rev1) [74,75,76,77]. Their proposed functions are the prevention of genetic and epigenetic instability at G-quadruplex sites, control of telomere length, as well as resolving quadruplex structures formed during DNA replication [78]. Unfortunately, to date, no structural data on quadruplexes bound by their protein partner are available.

## 3. Thermodynamic Consideration of DNA-Protein Interactions

In order to discuss the influence of DNA structure and sequence on the specific binding with a protein, interaction thermodynamics need to be considered. A protein has to bind significantly more to the recognition site than to competing, non-specific DNA. The equilibrium constant for non-specific binding ranges from 10^3^–10^6^ M^−1^ per site, with a free energy change of about −4 to −7 kcal/mole per site. Specific binding is 1000 to >10^7^ times tighter, but the affinity must not be excessive (<10^12^ per mole per site) since the binding needs to be reversible [79,80,81]. The gain in free energy in specific protein-DNA interactions is about −11 kcal/mole and per site. Chemical reactions and interactions only take place if the free energy ΔG_0_ is negative. Simplified, the free energy is the difference between the changes in enthalpy and entropy. Factors contributing positively to the change in enthalpy upon interaction are the formation of salt-bridges, non-polar contacts and hydrogen bonds. Nevertheless, the interaction has an entropic cost due to the loss of translational, rotational and configurational freedom and vibrations of entrapped waters. Enthalpically disadvantageous is the desolvatisation of the interface [82]. The net gain in enthalpy is further reduced by bond bending and unfavourable non-bonded interaction. Particularly, positioning of functional groups, residues and bases not at their lowest level of potential energy are disadvantageous. In addition, bending, distortion or destacking of the DNA increases the enthalpic cost. For instance, the TATA binding protein (TBP) unstacks 6 bases when binding to its respective promoter region, which costs 50–60 kcal/mol [83]. Generally, favourable changes in enthalpy are accompanied by an entropic penalty, while on the contrary favourable changes in entropy carry an enthalpic cost. For instance little or no distortion of the DNA is good for the enthalpic cost, but is unfavourable for the entropy change. The release of water from polar surfaces increases the entropy at an enthalpic cost. The transfer of a water molecule from bulk solution to a specific site carries an entropic penalty, which needs to be balanced by favourable van der Waals and hydrogen bonding interactions. Thus for the free energy to be negative, favourable enthalpy changes need to drive unfavourable entropy changes and *vice versa* [83,84].

## 4. Base Readout, Pre-Shaped DNA and Protein Recognition

DNA is a polyelectrolyte with high axial charge density and many counter ions/or water molecules at its surface. Therefore DNA binding proteins are often characterised by positively charged amino acids, such as arginine or lysine residues at the binding site for the interactions with the negative charged phosphate backbone. There are two mechanisms by which proteins specifically recognise a particular DNA: the base readout and the shape readout (reviewed in [85]). In the base readout mechanism, the specificity is achieved through direct or water mediated contacts with the DNA bases in the major and minor grooves. The major grove has the highest potential for base readout recognition since the functional groups of the four bases are displayed and are therefore accessible to specific interactions with the protein partner (A: 6-NH_2_, 7-N; C: 4-NH_2_; G: 6-O, 7-N; T: 4-O, 7-CH_3_). In contrast, only a two-letter code can be read in the minor groove (A: 3-N; C: 2-O; G: 3-N, 2-NH_2_; T: 2-O) (Figure 6). Therefore some sequences cannot be distinguished in the minor groove [86,87]. One also needs to take into account that bidentate H-bonds convey higher selectivity than single H-bonds [88]. In a number of protein-DNA complex structures, base specificity is mediated by conserved water molecules, which can be regarded as non-covalent extensions of the DNA bases [82]. An example for the use of waters in sequence-specific recognition is the Trp repressor-operator complex, which will be discussed below.

However, the sequence specificity for the majority of DNA binding proteins cannot be explained solely on the basis of specific protein-base contacts. For instance, transcription factors (TFs) achieve *in vivo* unique sequence specificity with seemingly identical DNA motifs but distinct targets. In a recent study Gordan *et al.* [89] demonstrated that the *Saccharomyces cerevisiae* bHLH TFs, Cbf1 and Tye1, bind their recognition sequence (E-box motif) depending on their genomic context. Further computational analyses suggest that nucleotides outside the E-box motifs contribute to specificity by influencing the three-dimensional structure of the DNA binding sites. Thus proteins evidently utilise the additional information from DNA structure and DNA deformability. In the example mentioned above, the bHLH TFs recognise the specific bases of the conserved E-box in the major groove, whereas local DNA shape recognition in the flanking regions appears to enable distinct DNA binding preferences among paralogous TFs [16,90].

**Figure 7:**
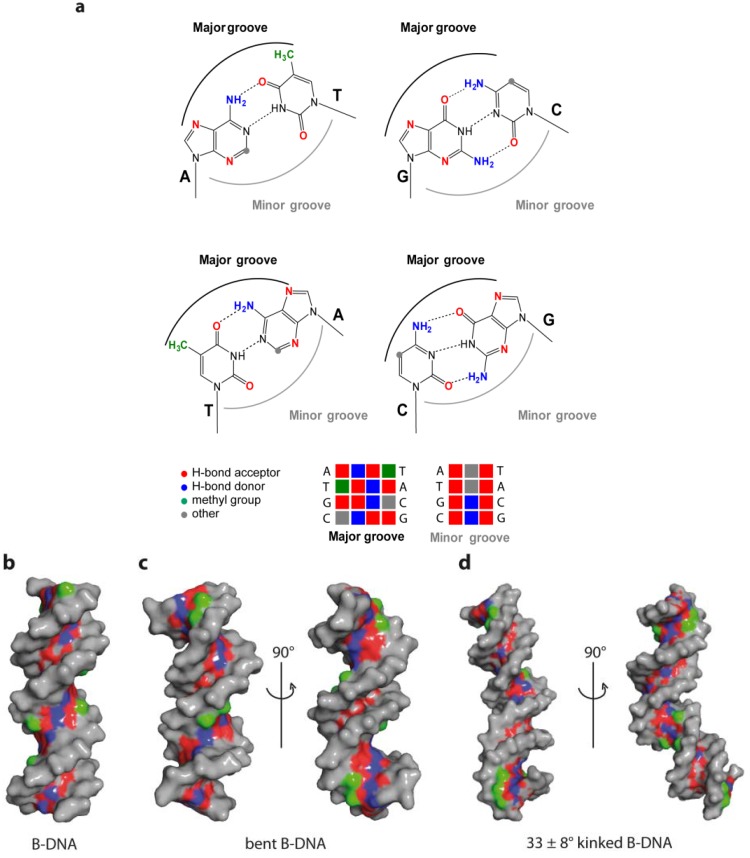
Base readout in the major and minor groove. (**a**) Functional groups of the DNA base pairs in the major and minor DNA groove; (**b**) Accessibility of functional groups in the major groove in a 16-mer B-DNA; (**c**) Locally increased readout accessibility in minor groove of bent B-DNA (PDB code 1JJ4); (**d**) Enlarged binding capability due to kink-induced shape alteration in B-DNA (PDB code 2KEI, [92]). Hydrogen bond donors in blue, acceptors in red and thymine methyl group in green.

The role played by the DNA sequence in this so-called shape readout mechanism can be explained through its influence on the conformational space predominantly occupied by a particular DNA as already described above. In this case a certain structure/conformation can be recognised and its stabilisation in the complex with the protein is thermodynamically favourable [91]. The shape readout mechanism can be further differentiated as local and global shape readout [85], depending whether the DNA deviation from B-DNA is local (kink) or more general (bend, A- or Z-DNA). However, one has to take into account that variation of DNA shape always influences the base readout: conformational changes of the DNA alter the geometry and hence accessibility of the bases in the major and minor grooves. Figure 7 highlights differences in the major and minor groove and therefore base readout options of B-DNA, bent DNA as well as kinked DNA.

In the following section, the recognition mechanisms will be illustrated in more detail, using structural examples of DNA-protein complexes. Therefore, the examples have been divided into: base readout (restriction endonuclease *Hin*dIII); base readout combined with intrinsic shape readout (restriction endonuclease *Eco*RV, Trp-repressor protein); sequence-context dependent base readout (LexA repressor); intrinsic shape readout (TATA binding protein); and overall shape readout (chromosomal protein HU, T7 endonuclease, ADAR1).

### 4.1. Base Readout and Recognition Sequence Intrinsic Shape Readout—The Restriction Endonucleases HindIII and EcoRV

**Figure 8:**
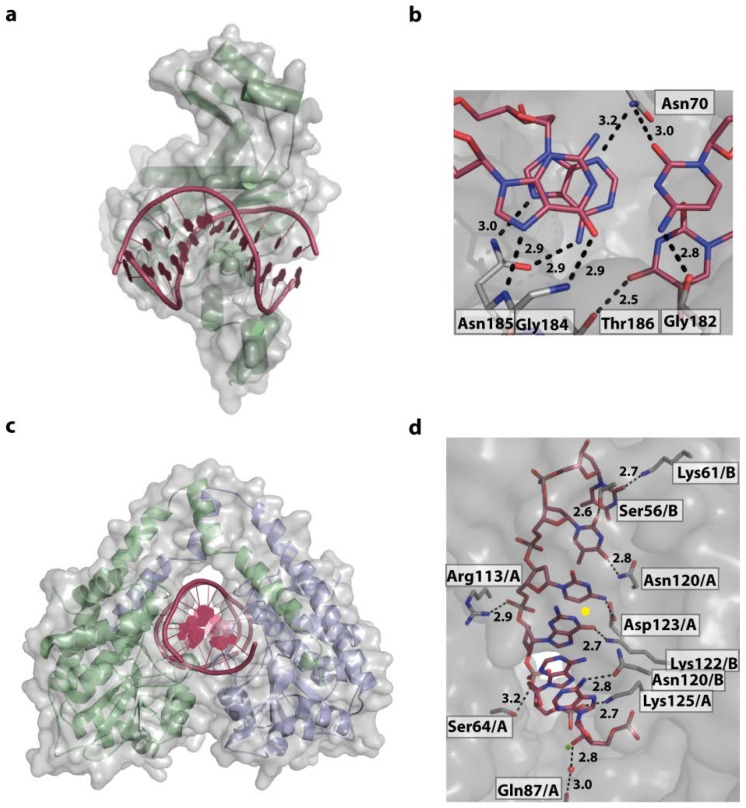
The restriction endonucleases *Eco*RV and *Hin*dIII. (**a**) Overall structure of the *Eco*RV DNA-complex (PDB code 1RVB). For clarity only one of two symmetrically binding protein molecules is shown. The DNA is kinked by 50°; (**b**) *Eco*RV interacts intensively with the outer GC and AT bases forming 8 direct contacts with almost all functional groups; (**c**) Structure of *Hin*dIII/DNA (PDB code 3A4K) complex displaying the symmetrical bound dimer embedding the DNA; (**d**) Active site of *Hin*dIII with, for clarity, one of the palindromic target sequence AAGCTT. Direct and water mediated contacts are formed. Water molecules, and for catalysis, required Mg^2+^ ions are shown as red and green spheres, respectively. The symmetry axis of the complex is located in middle of target sequence, between bases GC/CG and indicated as a yellow dot.

In the last century the discovery of bacterial restriction endonucleases has revolutionized molecular biology and numerous studies on their structures, mechanisms of action and DNA recognition are available to date. Bacteria have evolved restriction endonucleases as weapons against foreign DNA, such as viruses like bacteriophages [63,93,94]. These enzymes must be highly specific to cleave only DNA containing the respective recognition site, without degrading the host genomic DNA. Thus restriction endonucleases are an excellent, well investigated example for specific DNA sequence recognition in combination with enzymatic activity. They are grouped into four classes, according to: the nature of their target sequence; the cleavage position relative to the recognition sequence; co-factor; and structure. Type II restriction endonucleases cut DNA within or close to their palindromic recognition site [95], which they need to bind independent of the sequence context. Examples for classical type II endonucleases are *Hin*dIII from *Haemophilus influenza* and *Eco*RV from *E. coli*, which digest DNA at 5' A/AGCTT 3' and 5' GAT/ATC 3' sites, respectively. The structures of these enzymes in complex with their corresponding target DNA reveal that while *Hin*dIII relies predominately on direct base readout, *Eco*RV combines base- and local shape readout. Typical for type II endonucleases, two proteins matching the two fold rotational symmetry at the centre of the palindromic target sequence are bound to the DNA [96,97] (Figure 8). In the DNA-*Hin*dIII structure, both DNA strands and all six target bases located in the major groove are read out by the enzyme through 10 mono- and bidentate contacts, either directly or mediated by water molecules (Figure 8).

Two Mg^2+^-ions are located in the active site for catalysis. In addition, the structure clearly shows why methylation by *H. influenza* adenine methylases [98,99] protects against cleavage by *Hin*dIII: conversion of the first A of the target sequence to 6-met-A inhibits the interaction of Asn 120 with the 6-N amino group. In comparison *Eco*RV interacts intensively with the outer GC and AT bases forming 8 direct contacts with almost all functional groups [97] (Figure 8a,b). Outside the recognition motifs, both *Hin*dIII and *Eco*RV interact with the sugar-phosphate backbone. Common to all restriction endonucleases, they initially bind to DNA non-specifically and when their recognition site is encountered, enzyme and DNA undergo an induced-fit conformational change, resulting in DNA cleavage and release. The first weak non-specific DNA binding releases water and counterions from the DNA-protein interface. This balances the thermodynamically unfavourable loss of translational and rotational entropies upon complex formation that is further aided through the contacts made with the phosphate backbone, adding favourable enthalpy changes. Encounter with the cognate sequence triggers a cooperative conversion of the non-specific to the specific complex and couples sequence recognition to catalytic cleavage [95] The DNA bound by *Hin*dIII is slightly bent by about 26° in comparison to ideal B-DNA [96] (calculated with the program CURVES [44]). The target motif with the GC base step centrally located in the major groove reduces the local flexibility. This is due to the stiffness of the stacking purine-pyrimidine moieties as well as the steric hindrance by the 6-O of the G, reaching in the major groove. In contrast, the central, flexible AT base steps of the *Eco*RV target sequence (5' GAT/ATC 3') allows kinking by 50°. In summary, *Hin*dIII uses base readout on its whole target sequence, accompanied with a slight bend of the DNA. In comparison, *Eco*RV combines base readout by directly contacting a part of the target sequence with readout of the local shape provided by the flexible central AT sequence [100]. The induced-fit conformational change with the distortion of the DNA was shown to be essential for catalytic cleavage by type II restriction endonucleases. In structures of non-specific complexes neither the phosphate, or the catalytic residues nor the Mg^2+^ ions are positioned for cleavage [101]. Only upon DNA bending and structural changes of the enzymes the catalytic apparatus is assembled.

### 4.2. Combining Base and Shape Readout—The Escherichia coli Trp Operator

**Figure 9:**
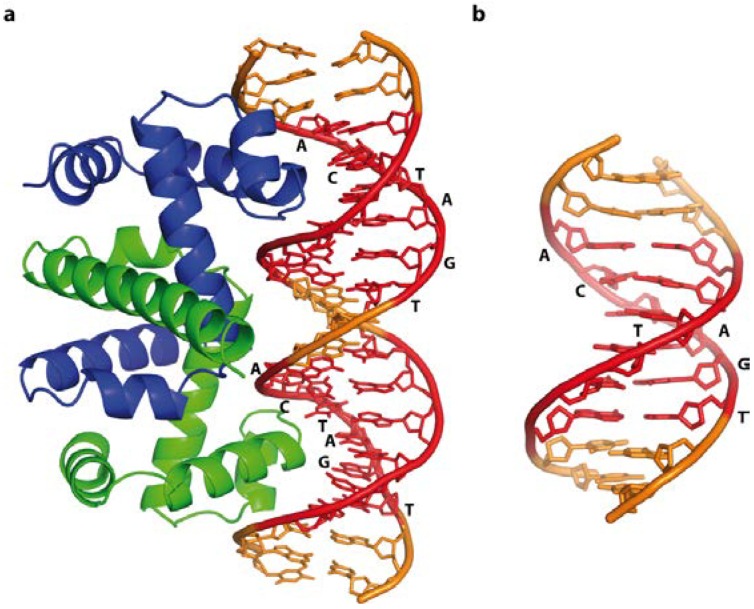
Free trp operator DNA, and in a complex with the Trp repressor protein, illustrating direct base readout and its impact on DNA structure. Cognate recognition sequences are highlighted in red. (**a**) Schematic representation of the trp operator-Trp repressor complex (PDB code 1TRO); The 15° bend compresses the major groove in the complex, whereas free DNA (**b**) (NDB code BDJ061) is straight, highlighting the subtle differences between bound and free DNA.

One of the first examples where structural data on a DNA sequence alone and in complex with a protein were available is the *E. coli* trp operator DNA and the Trp repressor protein [91,102]. This allowed dissecting the impact from the DNA sequence and its resulting structure on the specific recognition by its protein-binding partner. Comparing the structure of the free DNA and the DNA-protein complex revealed that specificity is conferred cooperatively by direct base readout, structural effects imposed by the DNA sequence and a large contribution by water-mediated interactions. Ten water binding sites in the major groove are conserved in the free and bound DNA of which three mediate specific contacts between the protein and nitrogen atoms of the purine bases. Replacing the purine nitrogens with a carbon atom resulted in a free energy difference of 1 kcal/mol on average, clearly showing that these water-mediated interactions are critical for the formation of a high-affinity complex [103]. While the free DNA is straight, the operator DNA in the protein complex is bent by 15°, resulting in compression of the major groove and widening of the minor groove. However both free and bound DNA are slightly unwound with 10.6 bases per turn and possess a deeper major groove, due to displacement of the base pairs from the helix axis. The observed displacement values of 0.9–1.9 Å are between A- and B-form DNA, albeit the backbone parameters are typical for B-DNA (Figure 9a). The structure adopted by the free trp operator DNA largely resembles its bound form even though it might be influenced by crystal packing forces (Figure 9b). The deformation energy barrier is therefore likely to be low, further facilitated by the central, more flexible T-A step [91,102]. This example clearly shows that both DNA shape driven by its sequence as well as specific base—amino acid interactions determine the specificity. 

### 4.3. Base Readout and DNA Shape Context—The Escherichia coli LexA Repressor

**Figure 10:**
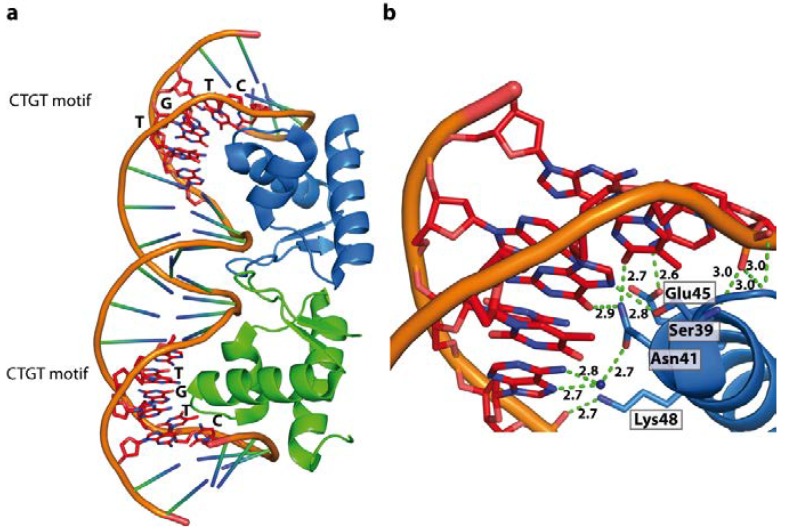
LexA repressor dimer bound to its cognate SOS boxes (PDB code 3JSO). (**a**) Schematic representation of LexA/DNA complex; (**b**) Extensive direct or water mediated contacts between the winged DNA binding domain and CTGT motif result in a 35° DNA bend towards the major groove.

The LexA repressor recognises the conserved CTGT motif (SOS-box), heavily relying on base readout [104]. However, the overlaying code of sequence-dependent DNA shape and flexibility by the flanking and interspacing sequences modulate binding affinity or determine whether a DNA-LexA complex is formed. The LexA repressor can therefore serve as a general model of how proteins achieve unique sequence specificity with seemingly identical DNA motifs, but distinct *in vivo* targets. In the LexA-DNA complex extensive direct or water-mediated contacts between the protein and the CTGT motif in the major groove are observed (Figure 10). The major groove is bent towards the protein resulting in an overall 35° curvature of the bound DNA. The entropic cost associated with DNA bending is compensated by contacts between the protein and the phosphate backbone in the adjacent regions. The impact of the flanking regions flexibility on the binding affinity has been shown, and the highest affinities were reported when the consensus sequence is flanked by TA and AT base steps (5'-TACTGT(AT)_4_ACAGTA-3') [105,106]. Due to the high flexibility of the AT steps, they can be placed in the narrow minor groove in a bent complex structure, since they provide only a low entropic penalty. Thus the LexA repressor clearly binds to its recognition motif by a combination of base-readout, where the affinity is driven by shape-readout of the flanking regions. This is further demonstrated by binding studies where the AT-spacer was replaced by less flexible DNA sequences such as A-tracts or G-C sequences, resulting in marked reduction of the binding affinity [104]. In a cellular context, decreased binding affinity caused by properties of the flanking regions is sufficient to determine whether or not a complex is formed. At first glance, the LexA and the Trp repressor appear to operate with a comparable recognition mechanism. However, while the flexibility of the flanking regions around the SOS-Box determines LexA binding, the Trp-operator sequence intrinsically encodes the DNA conformation recognised by the repressor protein.

### 4.4. Sequence Specific Shape Readout—The TATA Binding Protein and the TATA Box

**Figure 11:**
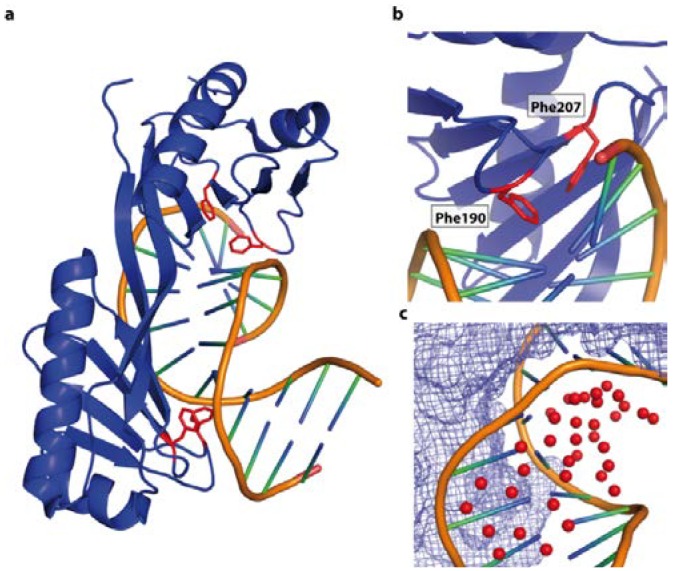
DNA shape recognition of the TATA box by the TBP binding (PDB code 1YTB). (**a**) Overall view of the complex. TBP is interacts with the minor groove, resulting in a compressed major groove. The contacts are dominated by van der Waals’ interactions occurring between nonpolar and polar atoms. The DNA is heavily unwound (105°) and displays two sharp kinks (highlighted in red); (**b**) Penetration of the double helix by Phe 190 and Phe 207 at the kink site; (**c**) The water molecules (red spheres) stabilize the major groove of the DNA. The protein surface is shown as blue mesh.

The previous examples illustrate that specificity can be governed by base readout, while the flexibility intrinsic to the target DNA or the sequence context impacts on the affinity or enables binding. In contrast, recognition of the TATA-box by the TATA binding protein (TBP) is predominately driven by the adopted shape of the DNA. The TBP plays a crucial role in transcription initiation by recognising the TATA-Box found in the −10 promoter region of many prokaryotic and eukaryotic promoters [107,108,109,110]. The structure of the heptameric TATA box recognition sequence bound to TBP (PDB code 1YTB) shows that the protein only contacts the minor DNA groove and no base specific contacts occur. The DNA in the complex is heavily unwound (105° over 7 bp), which is compensated by a superhelical turn of 120°. The minor groove is widened, a feature similar to A-DNA. The smooth overall 80° bend (90° in the central 6 bases) of the DNA is due to the additive effect of the large positive role angle of about 26° and the reduced twist of each base pair (Figure 11a). The compression of the major groove is stabilised by an extensive H-bond network and water molecules [111] (Figure 11c). In addition, pairs of phenylalanines penetrate the base pairs producing DNA kinks (Figure 11b). This indicates that the specificity is predominately driven by DNA shape [112]. The enthalpic cost for the bending of this DNA is lowered by the higher flexibility due to the reduced stacking of the T-A steps in the TATA-Box sequence, as observed for the trp operator. Further compensation is provided by the stabilisation through water-mediated and electrostatic interactions. Nevertheless the sequence flanking the TATA-box will have an impact on the overall stiffness and conformational space occupied by the DNA and hence on the binding affinity.

### 4.5. DNA Shape Recognition

#### 4.5.1. The Bacterial Chromosomal Proteins HU and IHF

**Figure 12:**
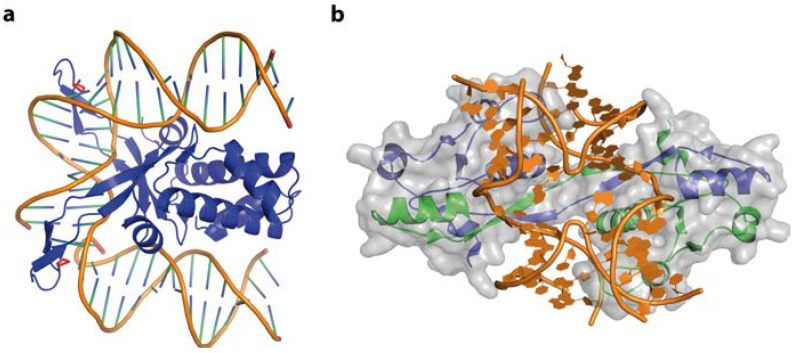
DNA-binding by IHF and T7 endonuclease I. (**a**) In the IHF-DNA complex (PDB code 1IHF) the DNA is roughly bent, with proline residues (red) intercalating, destacking the base pairs and stabilising the kinked structure. No direct or indirect contacts of IHF with the DNA major groove can be observed; (**b**) Illustration of a Holliday junction bound by T7 Endonuclease I (PDB code 2PFJ). The protein-DNA interactions results in large conformational changes: the duplex arms of the Hj are kinked by 80° and the junction centre is opened.

Further examples for DNA sequence driving DNA shape, with the resulting conformers being recognised rather than the bases by specific contacts, are the bacterial proteins HU (histone like protein from *E. coli* strain U93) and IHF (integration host factor). However the concept of sequence-dependent shape recognition in these examples is generalised, allowing the compensation for sequence variations. In the bacterial cell they are responsible for compacting the bacterial chromosome and maintenance of supercoiling and play an important role in DNA damage recognition, regulation of transcription and DNA replication [113,114,115,116,117,118,119,120]. Functionally, they are therefore homologous to the eukaryotic HMG box proteins [121,122]. HU and IHF have apparently no specific DNA target sequence, however it has been reported that HU, as well as IHF, bind tighter to A-tract [123], AT-rich sequences [124], pre-bent DNA, and DNA containing nicks or kinks [123,124]. This sequence preference was observed in a number of structural and biochemical studies on the protein-DNA complexes, which led to its thermodynamic explanation: both proteins bend the DNA about 105°–180°, base pairs within a 9 nucleotide distance are destacked, and the DNA is kinked twice. This base-destacking is accomplished by intercalating two conserved proline residues between the base pairs. No contacts with the major groove are observed and the DNA shape that is recognised has a large twist angle at the second dinucleotide step [125]. For IHF it could be shown that bending and tight specific binding is concerted [126], and it forces the two bends of the DNA to be almost coplanar (Figure 12a). The bend angles observed in HU-DNA complexes are less dramatic than in IHF-DNA complexes. However, the DNA has a negative writhe and is undertwisted by 2 bp compared to IHF (3 bp compared to B-DNA) [124]. Thermodynamically, the observed bending, twisting and kinking generates a large, unfavourable strain. DNA kinking alone was estimated to cost 14.1 kcal/mol [127]. How do IHF and HU compensate for this energetic penalty? Isothermal titration calorimetry (ITC) showed that binding of IHF to DNA is enthalpy-driven through the reorganisation and formation of surface salt bridges between protein and DNA [128]. In addition, binding to pre-bent DNA, which can be caused by DNA nicks and lesions or AT-rich sequences, correlates with a narrow minor groove that fits better in the protein clamp, at a lower enthalpic cost. Nevertheless, both proteins compensate the cost for nonspecific DNA binding and kinking by increasing the length of the binding site and formation of more salt bridges [124,129], a clear demonstration of the entropy–enthalpy compensation. This principle also applies to wrapping of DNA around nucleosomes in eukaryotic genome organisation events. Eukaryotic nucleosomes bind predominantly to so-called “positioning sequences” and with particular high affinity to the artificial “601” sequence [130]. This naturally deformable 601 sequence is a unique nucleotide repeating pattern: five TA base-pair steps (TA-TA dimers) roughly recurring in phase with every double-helical turn (~10 bp), are alternated with A + T-rich and G + C-rich motifs at the half-helical turns [131].

#### 4.5.2. The Holliday Junction and T7 Endonuclease I

*In vivo* Hjs are key intermediate of homologous recombination and DNA double-strand break repair. Their presence in negative supercoiled DNA was demonstrated to be essential for efficient plasmid replication, as well as genome stability [73,132,133,134]. Moreover, strong evidence linking transcriptional regulation and Hj DNA in promoter and enhancer regions has been found [135,136]. Numerous proteins involved in replication (helicases [137]), transcription (14-3-3 proteins [138,139,140]), DNA repair (XPG and XPF [141,142,143]), and chromatin remodelling (HMG box protein family [144,145,146]), are known to interact with Hj DNA. The proteins binding Hjs are diverse in their mechanisms and functions and have been reviewed extensively [147,148,149,150,151,152]. Structural data on Hj alone and in complex with their protein-binding partner are available [153,154,155]. In the next paragraph we want to focus on the role played by the Hj in the recognition event.

A well investigated example is the phage T7 endonuclease I (Endo I), which not only recognises and resolves Hjs but also branched DNA structures and, less efficiently, single-base heteroduplexes [156]. The efficiency was demonstrated to depend on the conformer population distribution. Nevertheless, it was shown that T7 Endo I only binds tightly to branched DNA duplexes where the duplex arms can adopt an 80° angle. When the crystal structures of the junction and the enzyme alone are compared with the complex, large-scale structural changes on both macromolecules can be seen [157]. In the junction, the B-form arms are still aligned coaxial, however the handedness of the junction is changed by a rotation of 130°, resulting in the 80° angle and an opened junction centre (Figure 12b). Extensive interactions of the junction backbone, in particular the arms that are subject to cleavage, with the basic protein surface are observed [157]. The enzyme does not directly interact with the DNA bases, albeit DNA sequence preferences in the junction arms exist [158]. The flexible N-terminal region (residues 1–16), which is not resolved in the crystal structure, was shown to stabilise the transition state to the active complex by locally opening the junction structure at the strand exchange points, thus reducing the activation energy by 3.8 kcal/mol [159].

Are there common principles how proteins interact with Hj? Generally they vary in their folding topology as well as the structure of the bound DNA. However, common to all Hj resolving enzymes is that they themselves show no direct sequence readout, but form extensive interactions with the sugar-phosphate backbone and appear to recognise the overall shape and conformational space occupied by Hjs in an induced-fit mechanism. Their dynamic character is crucial to allow moulding of the Hjs onto the generally large binding surface of predominately dimeric protein binding partners, at little energetic cost, ultimately leading to the observed tight binding (K_D_~1 nM) [158]. Since the same DNA junction can be bound by different enzymes, adopting distinct structures, this begs the question whether the protein binding partner imposes a shape upon the X-stacked structure, or traps a transient conformer of the free junction DNA. 

#### 4.5.3. Z-DNA and ADAR1

**Figure 13:**
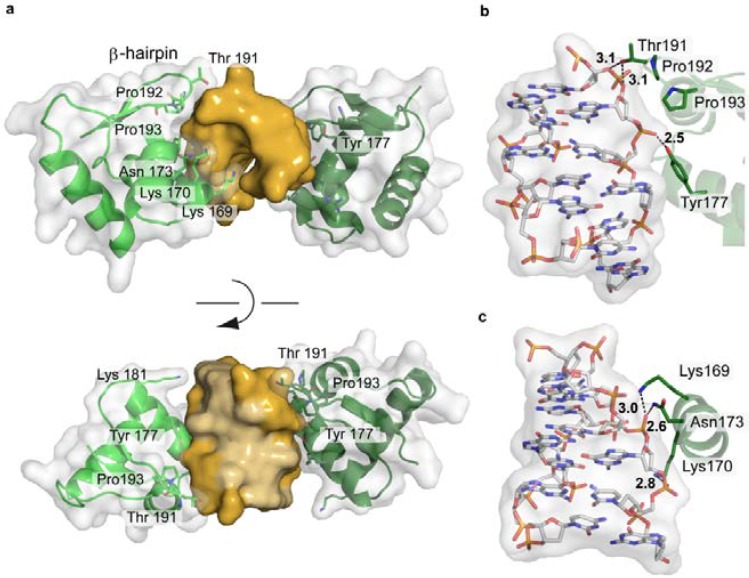
Structure of the Zα-domain of ADAR1 bound to left-handed Z-DNA (PDB code 3IRQ). (**a**) Schematic representation of the protein-DNA complex, highlighting the surface fit of the two macromolecules. The DNA is shown in gold, with the two bound proteins in green, overlaid with their semi-transparent surface; (**b**,**c**) Electrostatic interactions between ADAR1 and the Z-DNA backbone.

The IFN-induced form of the RNA editing enzyme ADAR1 deaminates adenine in pre-mRNA to inosine, which codes as guanine. However, its N-terminal Zα-domain is responsible for high-affinity binding to Z-DNA [160,161]. A biological function of Z-DNA binding by Zα has not been clearly defined yet. However Z-DNA is stabilized by negative supercoiling, which is formed transiently upstream of an active RNA polymerase [36]. Thus ADAR1 might be recruited to actively transcribed genes to act upon the nascent RNA. In addition, it was shown that ADAR1 edits viral genomes during viral transcription and alters viral growth [162]. The interaction between ADAR1 and Z-DNA is driven solely by the shape of the left-handed DNA, with the Zα-domain’s binding interface being complementary to the DNA in terms of conformation and electrostatic nature (Figure 13a). The Zα-domain has a HTH folding topology often found in proteins binding B-DNA. Nevertheless, the recognition modes are distinct, reflecting the different topologies of the DNA binding partner. While most HTH proteins use their helix α3 to interact with the major groove of B-DNA, helix α3 in ADAR1 only contacts the outer surface of the Z-DNA. The *C*-terminal β-hairpin of ADAR1 largely contributes to the binding [163]. The protein does not contact the DNA bases themselves, but forms an extensive hydrogen bonding network, both direct and water-mediated, to five consecutive backbone phosphates [163,164] (Figure 13b,c). In addition, van der Waals interactions between the DNA and cis-proline residues, located at the β-hairpin tip, and aromatic tyrosine side chains can be observed. (Figure 13). Thus a general DNA-recognising protein folding topology is adapted to match the shape of its DNA binding partner.

## 5. Conclusions

Rapid progress has been made in understanding how DNA structure functions as an overlaying code to the DNA sequence and its role in gene regulation, DNA damage recognition and genome stability. Biochemical, biophysical and structural studies on DNA and DNA-protein complexes have provided penetrating insights into how DNA sequence impacts on the structural and physical properties of this macromolecule and hence enables or prevents protein recognition. On the molecular level, whether a DNA protein complex is formed is determined by its free energy and the enthalpic and entropic gain and cost associated with each particular interaction. In general the DNA sequence determines and enables not only distinct interactions, but also the overall conformational space occupied by the DNA and therefore its shape. In complexes predominately driven by base specific readout, the DNA deviates little from its free conformation. In contrast, when there are few or no base-specific interactions between protein and DNA, one can observe that proteins recognise and stabilise DNA shapes, strongly diverging from the classical A- or B-DNA. Thus the finely balanced entropy—enthalpy compensation necessary for every interaction will be either rendered favourable and stable or unfavourable and instable.
